# Sustainable bioremediation of undiluted matured compost leachate and enhanced lipid partitioning in *Desmodesmus* sp.: a zero-dilution approach for circular bioeconomy

**DOI:** 10.3389/fmicb.2026.1872485

**Published:** 2026-08-26

**Authors:** Charith Akalanka Dodangodage, Jagath C. Kasturiarachchi, Thilini A. Perera, Sanjitha Dilan Rajapakshe, Sayuri S. Niyangoda, Rangika Umesh Halwatura

**Affiliations:** 1 ProGreen Lab, Department of Civil Engineering, University of Moratuwa, Moratuwa, Sri Lanka; 2 Department of Applied Sciences, Sri Lanka Institute of Information Technology, Malabe, Sri Lanka; 3 Department of Plant Sciences, University of Colombo, Colombo, Sri Lanka; 4 Department of Biosystems Technology, Uva Wellassa University, Badulla, Sri Lanka; 5 Department of Chemistry, University of Kansas, Lawrence, KS, United States

**Keywords:** *Desmodesmus* sp., lipid reprogramming, matured compost leachate, mixotrophic assimilation, zero-dilution bioremediation

## Abstract

The economic feasibility of microalgal biodiesel remains constrained by the high cost of synthetic nutrients, driving interest in waste-derived cultivation systems. This study investigates the mixotrophic growth of *Desmodesmus* sp. using 100% undiluted matured compost leachate as a dual-purpose medium for biomass production and wastewater remediation. To decouple microalgal assimilation from bacterial degradation, a strictly sterile abiotic control was employed, enabling accurate attribution of nutrient removal pathways. The system achieved bioremediation efficiencies of 82.6% chemical oxygen demand (COD) removal, alongside 60.3% nitrate reduction (via biological assimilation) and 65.5% phosphate reduction (via synergistic biological uptake and photosynthesis-induced physicochemical precipitation). *Desmodesmus* sp. demonstrated acclimatization to the untreated organic effluent, reaching a maximum biomass concentration of 2.69 g L^−1^—approximately fourfold higher than that obtained in the synthetic control medium. Progressive phosphorus exhaustion, driven by an extreme initial N:P ratio, induced lipid accumulation during the stationary phase, resulting in a lipid yield of 0.715 g L^−1^ (26.56% dry cell weight). Fatty acid methyl ester (FAME) analysis revealed a predominantly saturated profile (75.6%), dominated by palmitic and lauric acids, indicating a definitive metabolic shift from the highly unsaturated profile of baseline synthetic media. While such composition enhances oxidative stability, it may impose limitations on cold-flow properties. The detection of a non-saponifiable lipid fraction highlights a process bottleneck that must be addressed during downstream transesterification. This study demonstrates a zero-dilution, waste-to-resource platform that integrates high-density biomass production with nutrient recovery, offering a scalable pathway toward circular bioeconomy-driven biodiesel production.

## 1 Introduction

The rapid growth of the global population, accelerating urbanization, and expanding industrial activity are exerting unprecedented pressure on global energy systems, freshwater resources, and wastewater management infrastructures. Global population projections indicate an increase to approximately 9.7 billion by 2050, accompanied by an estimated 50% rise in global energy demand (Li and Li, 2023; Dodangodage et al., 2026a; Holechek et al., 2022). Despite growing renewable energy deployment, fossil fuels still account for nearly 80% of global primary energy consumption. This persistent dependence drives severe environmental degradation, with global carbon dioxide (CO_2_) emissions exceeding 35.8 Gt in 2023, intensifying climate change and air pollution (Liu et al., 2024). Among these impacts, fossil fuel-derived transportation fuels remain a major contributor to anthropogenic greenhouse gas emissions, underscoring the urgent need for sustainable, low-carbon liquid fuel alternatives (Shahzad and Iqbal Cheema, 2024).

Within this context, biodiesel has emerged as a promising renewable fuel due to its biodegradability, compatibility with existing diesel engines, and potential to reduce net greenhouse gas emissions. Global biodiesel consumption increased from approximately 2.2 million metric tons (MMT) in 2004 to 65.86 MMT in 2023 and is projected to reach nearly 75 MMT by 2030 (Awogbemi and Desai, 2025). However, the long-term sustainability of biodiesel production remains constrained by feedstock availability and cost. First-generation biodiesel derived from edible oils raises critical concerns related to food security, land-use change, and competition for agricultural resources, thereby motivating the development of non-food, third-generation biofuel pathways that decouple fuel production from arable land and conventional food systems (Rulli et al., 2016; Mhetras and Gokhale, 2025; Wang and Khanna, 2023).

Microalgae are widely regarded as one of the most promising third-generation biodiesel feedstocks owing to their rapid growth rates, high photosynthetic efficiencies, and superior lipid productivity compared to terrestrial oil crops (Casanova et al., 2023; Adewuyi, 2022; Behera et al., 2015; Zhang et al., 2022). Among various cultivation strategies, mixotrophic metabolism, where microalgae simultaneously utilize inorganic carbon (CO_2_) and organic carbon sources, has emerged as a highly efficient pathway for enhancing both biomass productivity and lipid accumulation. Under nutrient replete conditions, most microalgal species accumulate approximately 10–30% lipids (w/w), while nutrient stress, particularly nitrogen limitation, can enhance lipid accumulation by two- to three-fold (Maltsev et al., 2023; Udayan et al., 2023; Adams et al., 2013). Several chlorophycean strains, including *Desmodesmus*, *Chlorella*, and *Chlorococcum*, have been reported to achieve lipid contents exceeding 45–60%, while strategically enhanced lipid production via nutrient deprivation and photonic modulation has recently been demonstrated in emerging strains such as *Geminella* sp. and *Scenedesmus* sp. (Bhattacharjee and Baruah, 2025; Bhattacharjee and Baruah, 2026). In addition to high lipid yields, microalgal biomass grown under stress conditions often exhibits a favorable fatty acid methyl ester (FAME) profiletypically rich in saturated and monounsaturated fatty acids, making it an excellent candidate feedstock for potential renewable bioresource applications (Mallick et al., 2012; Mathimani et al., 2023).

Despite these advantages, the commercial deployment of microalgal lipid production remains economically challenging. The reliance on synthetic growth media, such as BG-11 and Bold’s Basal Medium (BBM), substantially increases production costs, with nutrient inputs alone accounting for approximately 30–50% of total cultivation expenses (Casanova et al., 2023; Gu et al., 2023). In contrast to synthetic media, which require the continuous purchase of analytical-grade nitrate and phosphate salts, matured compost leachate requires only simple physical pretreatment (filtration). At an industrial scale, this approach is expected to result in substantially lower operational costs compared to synthetic media (Maroušek et al., 2023; Rafa et al., 2021). Consequently, identifying low-cost, nutrient-rich alternative culture media has become a central research focus for improving the economic feasibility and scalability of microalgal systems.

In parallel, inadequate wastewater management continues to pose a major global sustainability challenge. Approximately 42–45% of household wastewater worldwide is discharged without safe treatment, while much of the remaining fraction receives only partial treatment, resulting in nutrient pollution, eutrophication, and widespread degradation of aquatic ecosystems (Staff, 2025; UN-Water and UN-Water, 2025). High-strength wastewaters enriched with nitrogen, phosphorus, and biodegradable organic matter therefore represent both an environmental burden and an underutilized resource. From a circular bioeconomy perspective, such waste streams offer substantial opportunities for nutrient recovery and bioresource generation when integrated with biological treatment processes (Dodangodage et al., 2025; Dodangodage et al., 2026b).

Among these waste streams, matured compost leachate wastewater, generated during the later stages of organic waste composting following prolonged biological stabilization, remains critically underexplored, particularly in the context of mixotrophic lipid biosynthesis. Compared to fresh leachates, which often contain inhibitory levels of volatile organic acids and heavy metals, matured compost leachate exhibits greater chemical stability and reduced concentrations of acute toxicants. While typical municipal wastewaters possess a relatively low organic load, and fresh landfill leachates present acutely toxic levels (often exceeding 20,000 mg L^−1^ COD) (Naveen et al., 2015), matured compost leachate occupies a strategic middle ground, providing abundant carbon without immediate lethality. Beyond mere cost reduction, this specific organic matrix is biochemically beneficial, offering pre-digested carbon substrates that integrate into microalgal metabolic pathways. These characteristics render matured compost leachate particularly suitable for biological treatment and valorization strategies, including microalgal cultivation (Ahamad Sanadi et al., 2021).

The integration of microalgal cultivation with wastewater treatment represents a compelling circular bioeconomy pathway by enabling simultaneous nutrient removal, biomass production, and renewable fuel generation. Wastewater-based microalgal systems have demonstrated effective reductions in chemical oxygen demand (COD), nitrate, and phosphate while producing lipid-rich biomass. More importantly, existing studies on compost-derived wastewaters have predominantly focused on treatment efficiency or biomass production in isolation. The literature is critically lacking an integrated evaluation of growth kinetics, nutrient removal, and lipid quality within a single system, particularly under mixotrophic conditions utilizing realistic, complex organic waste matrices. Specifically, the pure mixotrophic assimilation capacity of microalgae in complex organic waste matrices, and the subsequent impact of nutrient depletion on the metabolic restructuring of microalgal fatty acids, remain underexplored.

Notably, strain selection must prioritize not only high lipid accumulation but also tolerance to nutrient-rich and organically loaded environments. *Desmodesmus* sp. was selected in this study due to its demonstrated adaptability to realistic wastewater conditions, resilience to high nutrient concentrations, and capacity for lipid accumulation. Previous investigations have reported biomass productivities ranging from 0.2 to 1.8 g L^−1^ for *Desmodesmus* sp., alongside removals of 70–99% total nitrogen (TN), 60–95% total phosphorus (TP), and 40–80% COD, with lipid contents between 20 and 45% (w/w) and fatty acid profiles suitable for downstream applications (Ji et al., 2014; Zheng et al., 2023; Ogbonna et al., 2025; Mandotra et al., 2016).

This study is premised on the hypothesis that the simultaneous availability of light and organic carbon in matured compost leachate induces metabolic coupling between photosynthetic carbon fixation and heterotrophic assimilation pathways. Furthermore, we hypothesize that indigenous microbial consortia synergistically enhance organic carbon degradation, driving rapid nutrient depletion that subsequently triggers improved microalgal carbon partitioning toward lipid biosynthesis. Accordingly, the specific objectives of this study were to: (i) evaluate the growth kinetics of *Desmodesmus* sp. under varying leachate concentrations, including 100% undiluted raw leachate; (ii) quantify the simultaneous removal of COD, nitrate, and phosphate, employing a strict abiotic control to definitively differentiate the pure mixotrophic assimilation capacity of *Desmodesmus* sp. from baseline physical degradation; and (iii) analyze lipid accumulation dynamics and fatty acid profiling to mechanistically map the physiological response to nutrient stress. This study provides a mechanistic and process-level foundation for integrating mixotrophic microalgal cultivation with waste-derived nutrient streams, supporting circular bioresource utilization aligned with SDG 6, 7, and 12 (Sayago et al., 2024; Kanchanamala Delanka-Pedige et al., 2021; Dodangodage et al., 2026c,d; Fernando et al., 2026).

## 2 Materials and methods

### 2.1 Wastewater collection and pre-treatment

A composite sample of matured compost leachate wastewater (total volume: approximately 20 L) was collected from the secondary stabilization pond at the Karadiyana municipal solid waste dumping facility, South Colombo, Sri Lanka (6°48’57”N 79°54’11”E) during the inter-monsoonal dry season of 2025. To ensure a representative baseline, the wastewater was transported to the laboratory in sterile polyethylene containers at 4 °C and processed within 24 h. Standard Bold’s Basal Medium (BBM) (Bischoff, 1963) was used as the control without modifications. The preparation workflow consisted of physical filtration through a 0.45 μm nylon membrane (Millipore, United States) to remove suspended solids, initial physicochemical characterization, pH adjustment from its raw alkaline state (approximately pH 8.2) to 6.8 using 1 M HCl, and final sterilization via autoclaving (121 °C for 20 min) prior to microalgal inoculation. The clarified filtrate was subsequently used as the experimental growth medium.

### 2.2 Wastewater characterization

Initial physicochemical characterization of the matured compost leachate (including COD determination) was conducted in accordance with American Public Health Association (APHA) standard methods immediately after the pH was adjusted to 6.8 to accurately reflect the baseline conditions of the experimental growth medium. All measurements were performed using three independent biological replicates (*n* = 3 separate samples). The following parameters were analyzed (American Public Health Association, 1926):

- pH: Measured using a benchtop pH meter (SevenCompact, Mettler Toledo, Switzerland).
- Nitrate (NO_3_^–^-N): Measured using the dual-wavelength UV spectrophotometric method (APHA method 4500-NO_3_^–^ B) using a UV-Vis spectrophotometer (UV-1800, Shimadzu Corp., Kyoto, Japan). To eliminate optical interference from dissolved organic matter in the dark leachate matrix, absorbance was measured at both 220 nm and 275 nm, and the empirical correction formula (Abs_220_ − 2 × Abs_275_) was applied. Samples were strictly zeroed against a cell-free, filtered leachate blank at the corresponding dilution ratio prior to measurement.
- Phosphate (PO_4_^3−^–P): Ascorbic acid/molybdenum blue method, absorbance at 880 nm (APHA 4500-P E).
- Chemical Oxygen Demand (COD): Determined by the open reflux titrimetric method with potassium dichromate digestion (APHA 5220 B).

### 2.3 Microalgal strain and inoculum preparation

A pure culture of *Desmodesmus* sp. strain PGL-01 (maintained in the internal culture collection) was obtained from the ProGreen Laboratory, University of Moratuwa, Sri Lanka. This indigenous strain was selected for this study as it has previously demonstrated survival and adaptability in complex, toxic organic matrices. Pre-cultures were maintained in BBM containing standard nitrate conditions (250 mg L^−1^ NaNO_3_) under controlled laboratory conditions (25 ± 2 °C, continuous illumination at 150 μmol photons m^–1^ s^−1^ measured at the culture surface using cool-white LED lighting, and aeration at 0.5 vvm with sterile-filtered atmospheric air (≈0.04% CO_2_). Cells were harvested during the exponential growth phase by centrifugation at 3200 × g for 20 min (Eppendorf 5810R, Hamburg, Germany) and subsequently inoculated into experimental media at an initial biomass concentration of 0.30 g L^−1^. Inoculum was transferred directly from the BBM pre-culture to the experimental media without a prior stepwise acclimatization phase. This approach was chosen to directly evaluate the strain’s tolerance and metabolic adaptability to the raw leachate. Cultivation experiments were conducted in 2 L laboratory-scale glass photobioreactors with a working volume of 1.5 L. Each reactor was equipped with three-port GL45 screw caps to facilitate aeration, sampling, and pressure compensation. Aeration ports were fitted with 0.45 μm polytetrafluoroethylene (PTFE) membrane filters to ensure sterility and minimize evaporative losses. Illumination was provided using cool-white LED strips under a 12:12 h light–dark photoperiod (Dodangodage et al., 2026e; Naseema Rasheed et al., 2023; Nicodemou et al., 2024).

### 2.4 Experimental setup and cultivation conditions

#### 2.4.1 Phase I: screening of dilution ratios

Screening experiments were conducted using four dilution ratios of matured compost leachate wastewater prepared using distilled water: 25% (100 mL leachate + 300 mL distilled water), 50% (200 mL leachate + 200 mL distilled water), 75% (300 mL leachate + 100 mL distilled water), and 100% undiluted leachate (400 mL leachate + 0 mL distilled water). Cultures were grown in 500 mL Erlenmeyer flasks containing a working volume of 400 mL. BBM (400 mL) served as the control medium. The calculated initial physicochemical properties and nutrient loads of these five treatment media are detailed in section 3.1.

The initial pH of all treatments was adjusted to 6.8 using NaOH. Cultures were incubated at 25 ± 2 °C under a 12:12 h light–dark cycle, continuously aerated, and illuminated at an intensity of 150 μmol photons m^–1^ s^−1^ measured at the culture surface using cool-white LED lighting. Biomass growth was monitored at 48 h intervals by measuring optical density at 680 nm (OD_680_) and by dry weight determination.

#### 2.4.2 Phase II: physiological characterization and lipid production

Based on Phase I results, 100% undiluted matured compost leachate (100% v/v), which demonstrated optimal growth performance and substrate availability, was selected for the main cultivation experiment. To rigorously evaluate the underlying mechanisms of nutrient and COD reduction, batch cultivation was performed in 2 L sterilized glass photobioreactors (1.5 L working volume) using two distinct configurations: (1) Experimental Culture: Autoclaved leachate inoculated with *Desmodesmus* sp.; and (2) Abiotic Control: Autoclaved leachate without microalgal inoculation, designed to quantify baseline physical or chemical degradation. BBM inoculated with *Desmodesmus* sp. served as the baseline growth control.

While the screening phase was conducted over 20 days to map early exponential growth dynamics, a 24-day cultivation period was selected for the main experiment to ensure the culture fully traversed the exponential phase and entered the stationary phase, which is critical for lipid accumulation. Cultures were maintained at 25 ± 2 °C under a fixed 12:12 h light–dark cycle, aerated at a constant rate of 0.5 vvm with sterile-filtered atmospheric air (≈0.04% CO_2_; BOYO pumps, China; 0.45 μm filters), and illuminated at a constant intensity of 150 μmol photons m^–1^ s^−1^ measured at the reactor surface using cool-white LED lighting. Light intensity was periodically verified using a quantum sensor (LI-COR LI-250A, United States).

### 2.5 Analytical procedures

#### 2.5.1 Biomass concentration

Biomass measurements (OD_680_ and DCW) were exclusively performed on the inoculated experimental cultures and the baseline BBM control. The abiotic control was not subjected to biomass tracking, as it was designed solely to isolate liquid-phase nutrient and COD degradation. Microalgal growth was monitored every 48 h, with additional daily sampling implemented during the late stationary phase (days 20–24) to accurately capture peak biomass accumulation. Optical density at 680 nm (OD_680_) was measured using a UV–Vis spectrophotometer (Shimadzu UV-1800). To account for the high background absorbance of the dark, turbid leachate, the spectrophotometer was strictly zeroed using a blank of cell-free, filtered matured compost leachate at the corresponding dilution ratio prior to each measurement.

However, due to the inherent optical interference of the wastewater matrix, gravimetric Dry Cell Weight (DCW) was employed as the primary and definitive metric for biomass assessment. Biomass concentration was determined gravimetrically by filtering 5 mL culture aliquots through pre-dried and pre-weighed glass microfiber filters (GF/C, 1.2 μm pore size, Hyundai Micro, South Korea) (Dodangodage et al., 2026f). Filters were oven-dried at 60 °C for 24 h prior to weighing. Biomass concentration (DWn, g L^−1^) was calculated as:


DryWeight(gL)-1(DWn)=Wf-WiV

where *W_f_* is the final weight of the filter containing the dried biomass (g), *W_i_* is the initial weight of the pre-dried empty filter (g), and *V* is the volume of the filtered culture sample (L). In this study, “Dry Biomass Accumulation” refers to the total harvested biomass per liter of culture volume (used for yield comparison), whereas “Dry Cell Weight (DCW)” represents the temporal measurement of biomass density during the growth curve.

#### 2.5.2 Nutrient and COD removal

Nitrate, phosphate, COD, and pH were measured at 2-day intervals. Liquid samples (5 mL) from both the experimental cultures and the abiotic controls were centrifuged at 10,000 × g for 5 min, and the supernatant was filtered through 0.22 μm nylon syringe filters. Nitrate and phosphate concentrations were analyzed as described in section 2.2, while pH was measured using a calibrated pH probe (Hanna HI98194) (Dodangodage et al., 2026f). Removal efficiency (RE, %) was calculated as:


$$\left(R\,E\right)=\frac{C_{i}-C_{f}}{C_{i}}\times 100$$

where *C_i_*and *C_f_*represent the initial and final concentrations (mg L^–1^) respectively. To strictly isolate the specific mixotrophic assimilation capacity of *Desmodesmus* sp., the net biological COD removal was evaluated by comparing the total reduction in the experimental culture against the baseline physical degradation observed in the abiotic control.

#### 2.5.3 Analytical validation

All analytical measurements were conducted in triplicate. UV–Vis analyses were calibrated using standard solutions of potassium nitrate, KH_2_PO_4_, and potassium hydrogen phthalate. Calibration curves demonstrated strong linearity (R^2^ ≥ 0.996). Instrumental blanks were analyzed with each batch to minimize baseline drift.

#### 2.5.4 Growth kinetics modeling

To accurately describe the non-linear growth dynamics of the microalgae, the rate of biomass accumulation was initially defined by the differential logistic growth model:


$$\frac{d\,X}{d\,t}=\mu_{m\,a\,x}\,\left(1-\frac{X}{X_{m\,a\,x}}\right)\,X$$

Integration of this equation yields the standard logistic model, which was applied for the non-linear regression fitting of the biomass data (DCW):


$$X=\frac{X_{m\,a\,x}}{1+\left(\frac{X_{m\,a\,x}}{X_{0}}-1\right)\,e^{-\mu_{m\,a\,x}\,t}}$$

where *X*is the biomass concentration (g L^–1^) at cultivation time *t*(days), *X_0_*is the initial biomass concentration (g L^–1^), μ_*max*_is the maximum specific growth rate (d^–1^), and *X*_*max*_is the maximum carrying capacity (g L^–1^). The model was fitted using the integrated logistic equation to obtain μ_*max*_and *X*_*max*_.

Additionally, to evaluate the temporal metabolic variations during the cultivation period, the interval-based specific growth rate (μ, d^–1^) was calculated between successive sampling points using the equation:


$$\mu =\frac{\ln\,\left(X_{2}\right)-\ln\,\left(X_{1}\right)}{t_{2}-t_{1}}$$

where *X_2_*and *X_1_*are the dry cell weight concentrations (g L^–1^) at times *t_2_*and *t_1_*, respectively.

### 2.6 Biomass harvesting

At the end of the 24-day cultivation period, microalgal biomass was harvested by centrifugation at 4,500 × g for 10 min (Eppendorf 5810R). The biomass was washed twice with sterile distilled water and oven-dried at 60 °C until constant weight was achieved (Dodangodage et al., 2025; Potts, 2000).

### 2.7 Lipid extraction and quantification

Lipid extraction and subsequent FAME profiling were exclusively conducted on the inoculated experimental cultures. The abiotic control was excluded from these procedures due to the absence of microalgal biomass. Using the dried biomass harvested in section 2.6, lipid extraction was performed using a modified Bligh and Dyer protocol (Bligh and Dyer, 1959). Briefly, 10 mg of dried biomass was homogenized using vortex mixing for 5 min with a chloroform–methanol mixture (1:1, v/v) to facilitate lipid solubilization. Phase separation was subsequently induced by adding 0.8% (w/v) NaCl solution, resulting in a final solvent ratio of chloroform/methanol/0.8% NaCl = 10:10:9 (v/v/v).

The lower chloroform phase containing the extracted lipids was carefully transferred into pre-dried and pre-weighed centrifuge tubes and evaporated under a gentle stream of nitrogen gas. Residual solvent was removed by oven drying at 60 °C until constant mass was achieved. The total lipid content was quantified gravimetrically and expressed as a percentage of dry biomass (Lee et al., 2013; Andrade et al., 2021).

### 2.8 Fatty acid methyl ester (FAME) preparation and fatty acid profiling

Total lipids extracted as described in section 2.7 were dissolved in chloroform, and 0.5 mg of heptadecanoic acid (C17:0) was added to each sample as an internal standard prior to solvent evaporation under nitrogen. Fatty acid transesterification was conducted by adding 5 mL of 1% (v/v) sulfuric acid in methanol, followed by incubation in a 50 °C water bath overnight, as previously reported.

The resulting fatty acid methyl esters (FAMEs) were extracted into n-hexane and analyzed using gas chromatography with flame ionization detection (GC–FID) (7890A, Agilent Technologies, United States) equipped with a DB-23 capillary column (60 m × 0.25 mm × 0.15 μm). The injector temperature was maintained at 250 °C, with an injection volume of 1 μL and a 1:50 split ratio. Helium was used as the carrier gas at a constant pressure of 230 kPa (Breuer et al., 2013; Ma et al., 2016).

The oven temperature program consisted of an initial hold at 50 °C for 1 min, followed by an increase at 25 °C min^−1^ to 175 °C, then ramped at 4 °C min^−1^ to 230 °C and held for 5 min. The detector temperature was set at 280 °C, with hydrogen, air, and helium flow rates of 40, 450, and 30 mL min^−1^, respectively. Fatty acids were identified and quantified using a Supelco 37-component FAME standard mixture, which was used for calibration.

### 2.9 Statistical analysis

All experiments, including the abiotic controls, were conducted in independent biological triplicates, and results are expressed as mean ± standard deviation (SD). Data normality and homogeneity of variance were assessed prior to statistical testing. Differences among treatment groups concerning final biomass yields, lipid content, and total nutrient removal were evaluated at the conclusion of the cultivation period (day 24) using a one-way analysis of variance (ANOVA). The significance level was set at α = 0.05. When significant main effects were detected (*p* < 0.05), Tukey’s *post-hoc* test was applied for multiple comparisons among the treatment groups. Statistical analyses were performed using Minitab 17, and graphical representations were generated using Microsoft Excel (Office 365).

## 3 Results

### 3.1 Characterization of matured compost leachate wastewater

The physicochemical characteristics of the pH-adjusted matured compost leachate collected from the Karadiyana dump site are summarized in Table 1. The leachate exhibited a slightly acidic to near-neutral pH (6.83 ± 0.15) and a dark brown, turbid appearance, indicating the presence of dissolved humic substances and stabilized organic matter.

**TABLE 1:** Physicochemical characterization of raw matured compost leachate wastewater used in the study.

Parameter	Unit	Value (Mean ± SD)
pH	–	6.83 ± 0.15
Chemical Oxygen Demand (COD)	mg L^−1^	1365.89 ± 48.19
Nitrate (NO_3_^–^–N)	mg L^−1^	735.29 ± 17.87
Phosphate (PO_4_^3−^–P)	mg L^−1^	20.87 ± 1.20
Appearance	–	Dark brown, turbid

The organic load was considerable, with a Chemical Oxygen Demand (COD) of 1365.89 ± 48.19 mg L^−1^. However, when compared to fresh compost or landfill leachates, which can exhibit COD levels in the range of 10,000–50,000 mg L^−1^, this value supports the matured nature of the effluent, reflecting partial biological stabilization during composting. This moderate carbon load provides a favorable substrate for mixotrophic microalgal metabolism without inducing acute organic toxicity.

Nutrient analysis revealed elevated concentrations of nitrate and phosphate, classifying the leachate as a high-strength wastewater. Nitrate (NO_3_^–^–N) and Phosphate (PO_4_^3−^–P) concentrations were 735.29 ± 17.87 and 20.87 ± 1.20 mg L^−1^, respectively. The resulting N:P ratio (∼35:1) indicates a phosphorus-limited nutrient balance. Despite this imbalance, the high nutrient content highlights the potential of this waste stream as a nutrient-rich cultivation medium, provided the microalgal strain can tolerate the associated turbidity and high nitrogen loading. The estimated initial nutrient and organic carbon compositions for all screening media (Media 1–5) are detailed in Table 2.

**TABLE 2:** Estimated initial nutrient and organic carbon composition of the screening media (Media 1–5) based on dilution ratios.

Treatment	Dilution (v/v)	COD (mg L^−1^)	Nitrate (NO_3_^–^–N) (mg L^−1^)	Phosphate (PO_4_^−3^–P) (mg L^−1^)	Estimated N:P ratio
Media 1	25% Leachate	∼ 341.5	∼ 183.8	∼ 5.2	∼ 35:1
Media 2	50% Leachate	∼ 682.9	∼ 367.6	∼ 10.4	∼ 35:1
Media 3	75% Leachate	∼ 1024.4	∼ 551.5	∼ 15.7	∼ 35:1
Media 4	100% Leachate	1365.89	735.29	20.87	∼ 35:1
Media 5	BBM (Control)	0	139.37	7.37	∼ 19:1

### 3.2 Effect of leachate dilution on microalgal growth (screening phase)

The growth response of *Desmodesmus* sp. was evaluated under varying leachate dilutions (25, 50, 75, and 100% v/v) and compared with a BBM control over a 20-day cultivation period. One-way ANOVA revealed statistically significant differences in growth performance among treatments based on the endpoint measurements recorded on day 20 for both optical density (*p* < 0.001) and dry cell weight (*p* < 0.001).

**FIGURE 1:**
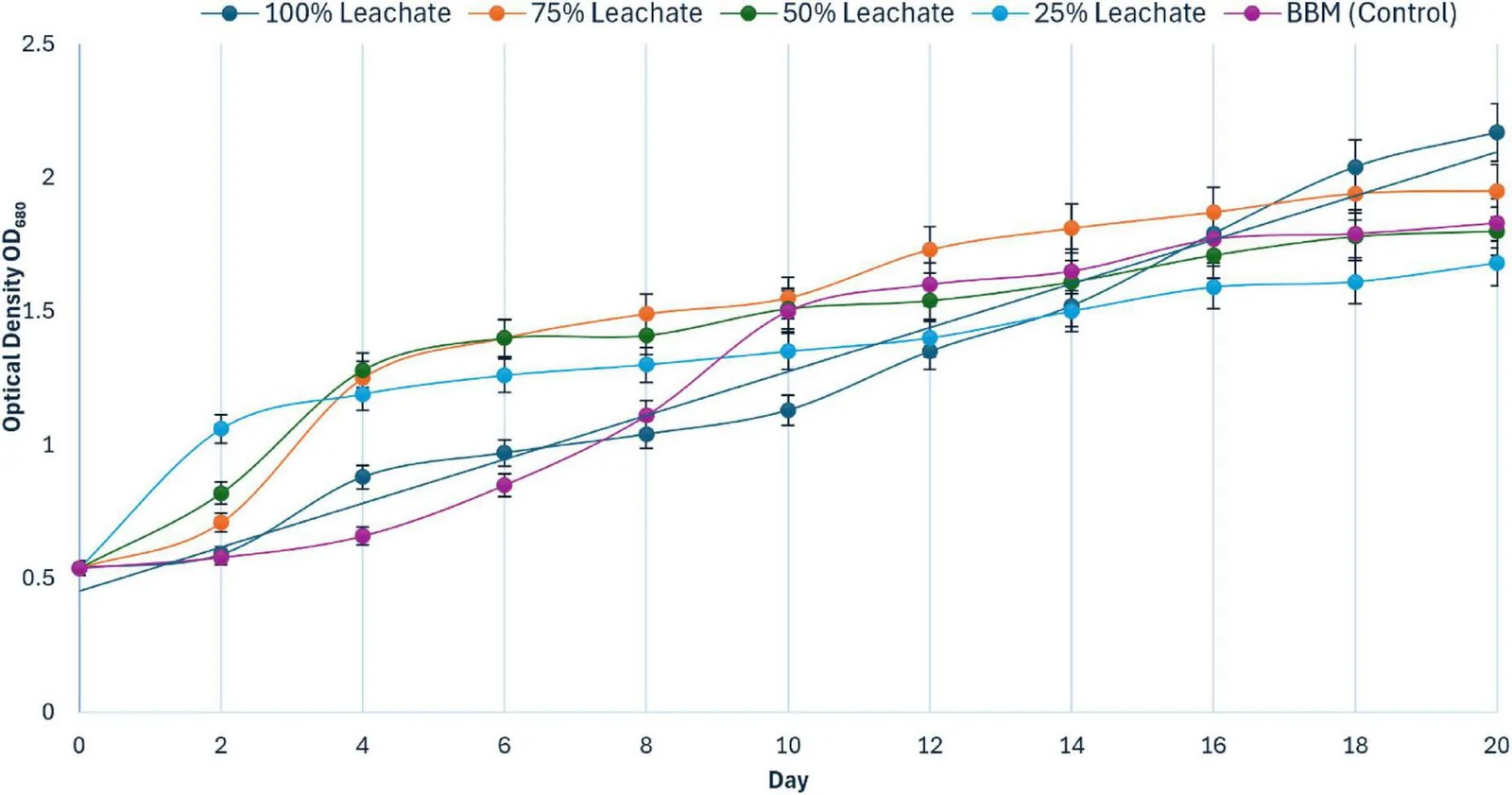
Time-course optical density (OD680) growth profiles of *Desmodesmus* sp. under varying leachate dilutions (25, 50, 75, 100% v/v) compared with the BBM control over a 20-day cultivation period. Error bars represent standard deviation (*n* = 3).

As shown in Figure 1, cultures grown in higher leachate concentrations (75 and 100%) exhibited a distinct lag phase during the first 4 days, whereas lower dilutions and the control showed earlier growth initiation. Following this initial period, rapid exponential growth was observed in all leachate treatments.

To accurately capture these non-linear growth dynamics, the biomass accumulation data was fitted to a Logistic growth model. For the 100% leachate condition, the fitted model yielded a maximum specific growth rate (μ_*max*_) of 0.152 d^−1^ and a carrying capacity (X_*max*_) of 1.714 g L^−1^, (with an R^2^ of 0.96) indicating strong model agreement with experimental data.

**FIGURE 2:**
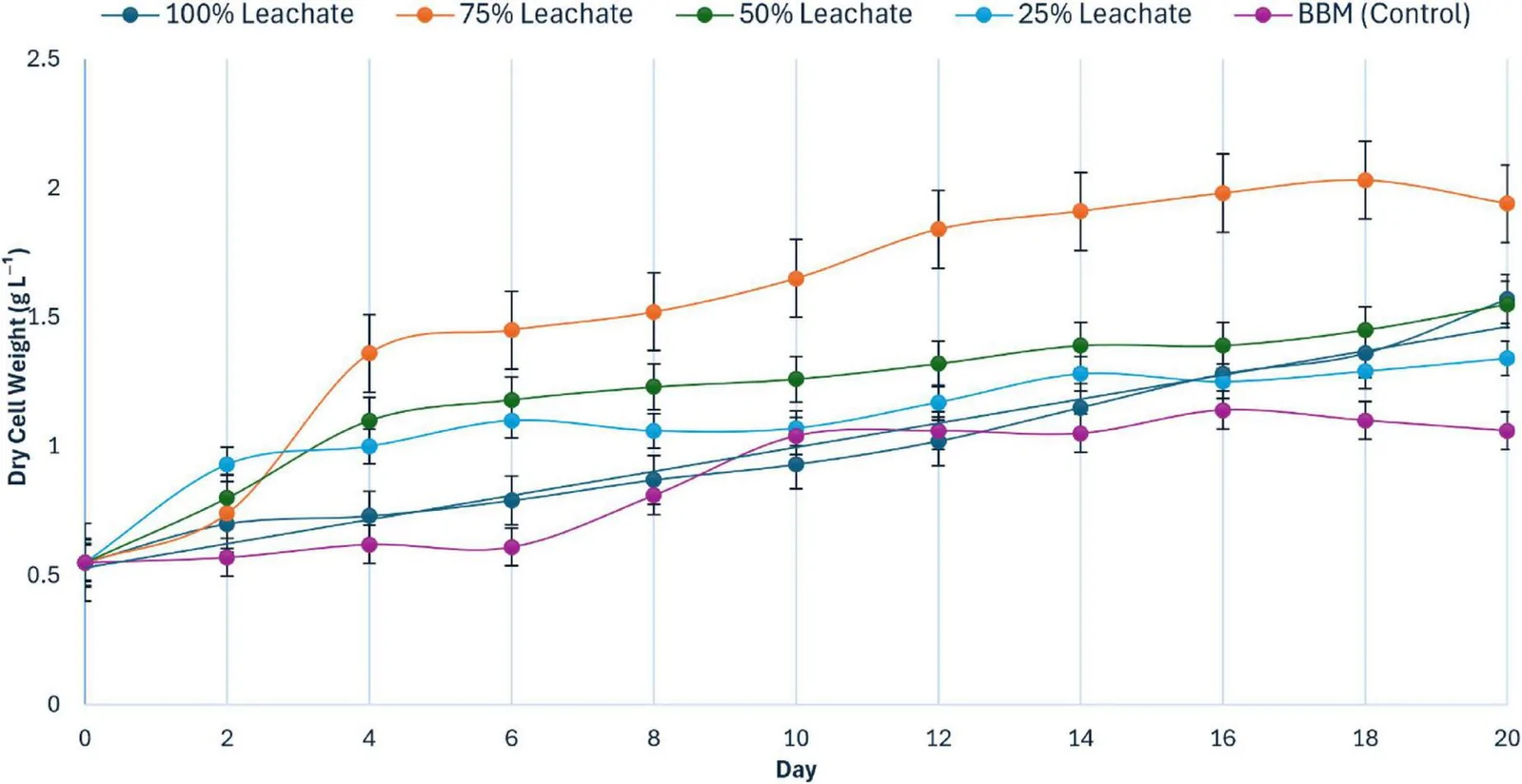
Time-course dry biomass accumulation of *Desmodesmus* sp. across different leachate dilutions and the BBM control over a 20-day cultivation period. Error bars represent standard deviation (*n* = 3).

By day 20, cultures in undiluted leachate (100%) achieved the highest optical density (2.269 ± 0.072), which was significantly higher than both the BBM control (1.668 ± 0.070) and the 75% dilution (1.894 ± 0.003) (*p* < 0.05). However, gravimetric dry biomass analysis (Figure 2), thereby bypassing optical interference caused by the dark wastewater matrix, revealed that the 75% leachate yielded the highest absolute dry biomass (2.025 ± 0.089 g L^−1^). Importantly, the undiluted leachate still produced substantially higher biomass (1.567 ± 0.072 g L^−1^) than the synthetic BBM control (1.063 ± 0.048 g L^−1^).

Based on these findings, the 100% undiluted leachate condition was selected for subsequent Phase II experiments to evaluate the microalgal tolerance and maximum bioremediation potential under raw, unmitigated wastewater conditions.

### 3.3 Biomass production in main cultivation experiment

**FIGURE 3:**
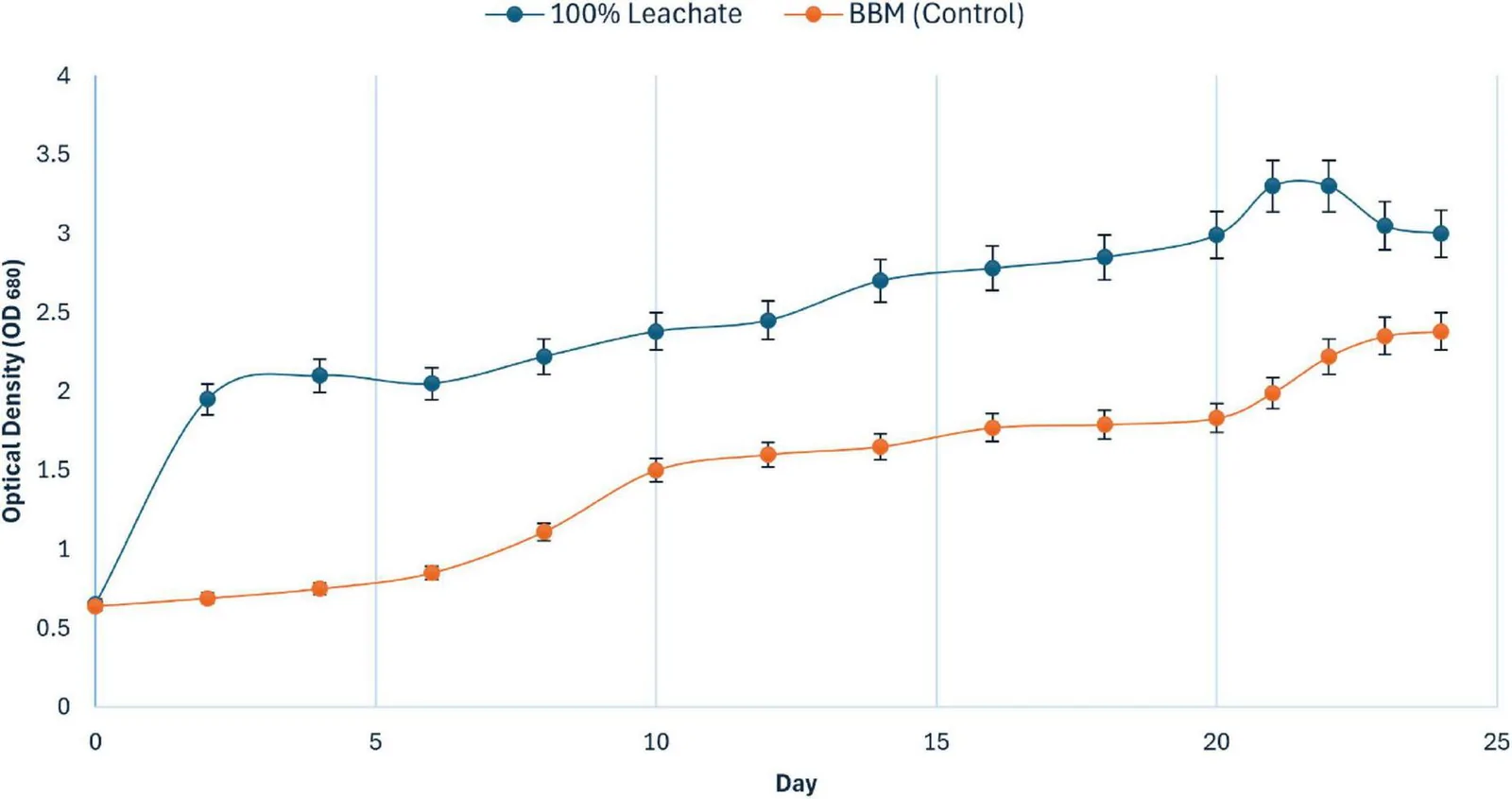
Time-course optical density (OD_680_) growth kinetics of *Desmodesmus* sp. in undiluted matured compost leachate and the BBM control over a 24-day batch cultivation period. Error bars represent standard deviation (*n* = 3).

Growth kinetics of *Desmodesmus* sp. were further evaluated in undiluted matured compost leachate and BBM over a 24-day batch cultivation period. The 100% undiluted leachate initially yielded lower absolute dry biomass in the screening phase due to a transient shock induced by high ammoniacal nitrogen concentrations and dark-matrix toxicity. However, the step-wise scale-up into the 1.5 L main bioreactor provided extended acclimatization time, allowing the *Desmodesmus* sp. cells to upregulate necessary detoxification pathways (such as antioxidant enzyme activation) (Figure 3).

**FIGURE 4:**
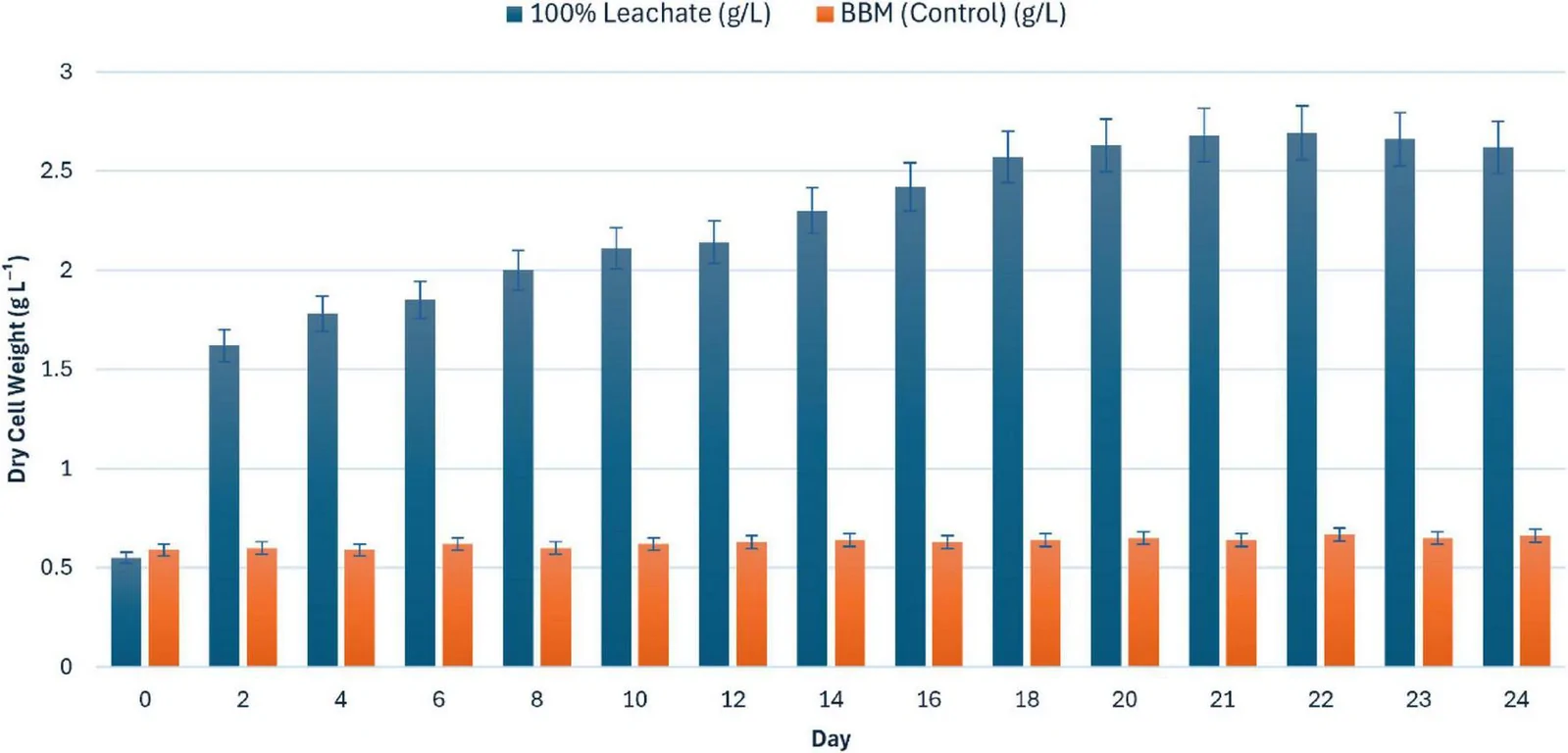
Dry cell weight (DCW) of *Desmodesmus* sp. in matured compost leachate versus BBM control during 24 days of batch cultivation.

Given the potential influence of wastewater turbidity and cellular morphology on optical measurements, biomass productivity was assessed using dry cell weight (DCW). As shown in Figure 4, DCW values in the leachate culture were consistently and significantly higher (p < 0.001) than those in the control throughout the cultivation period. The maximum biomass concentration in leachate reached 2.692 ± 0.093 g L^−1^ on day 22, compared to only 0.668 ± 0.031 g L^−1^ in the BBM control.

The cultivation in matured compost leachate resulted in a fourfold higher biomass accumulation relative to synthetic media, demonstrating its suitability as an effective growth substrate for high-density algal cultivation. To further characterize the cultivation performance in the 100% matured compost leachate, key kinetic parameters were determined for the main experimental phase: the maximum biomass yield (X_*max*_) reached 2.69 g L^−1^, the total biomass yield achieved was 2.14 g L^−1^, the maximum specific growth rate (μ_*max*_) was modeled at 0.152 d^−1^, and the overall biomass productivity (P) averaged 0.097 g L^−1^ d^−1^.

**FIGURE 5:**
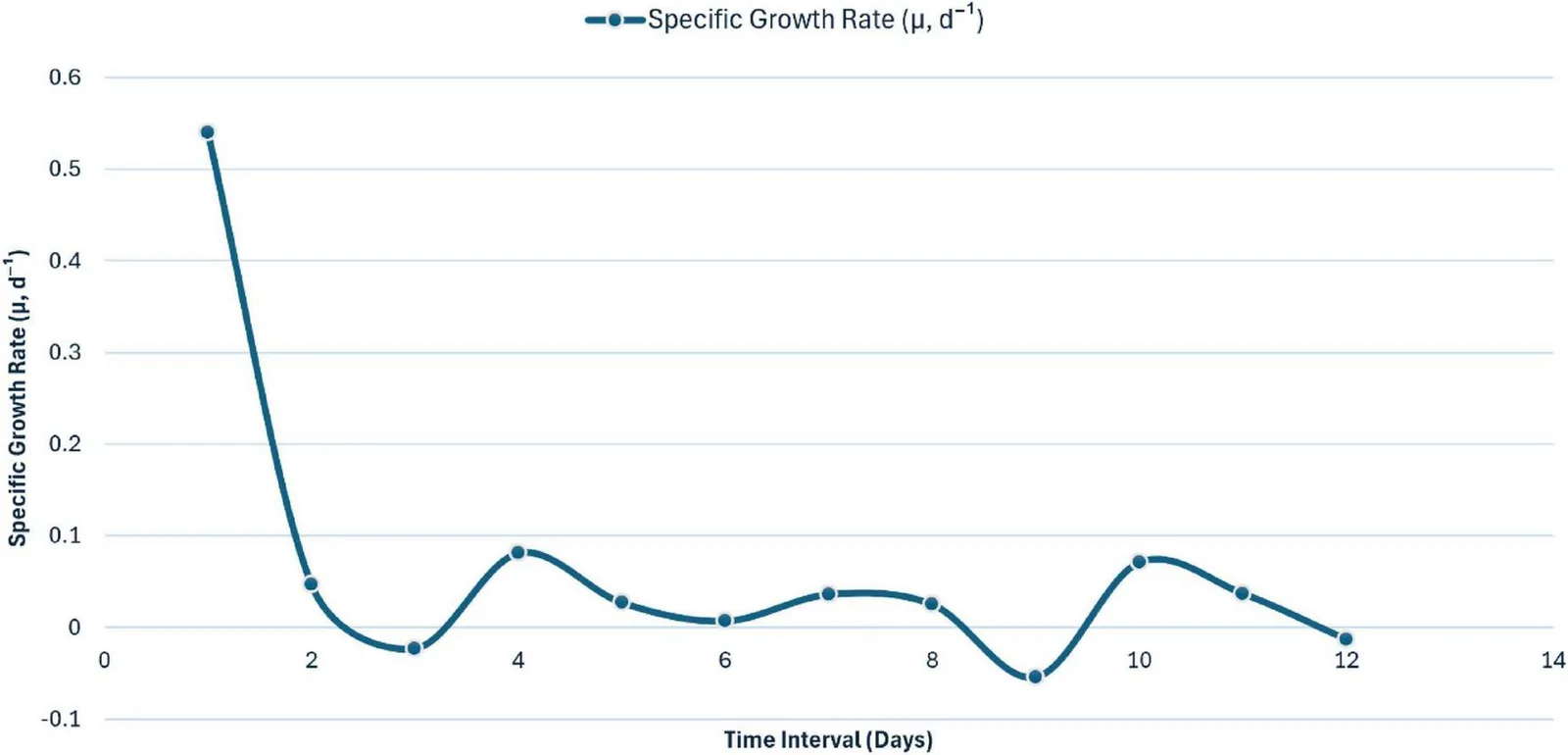
Discrete interval-based specific growth rates of *Desmodesmus* sp. cultivated in 100% matured compost leachate over the 24-day period. Error bars represent standard deviation (*n* = 3).

The temporal variation of the specific growth rate during this period is illustrated in Figure 5. The interval-based calculation reveals a metabolic acceleration immediately following the initial acclimatization phase, peaking transiently at ∼0.54 d^−1^ at the earliest interval, before gradually declining as culture density increases and nutrient availability changes. This 0.54 d^−1^ value represents a localized, short-term exponential burst. In contrast, the globally fitted logistic maximum specific growth rate (μ_*max*_) of 0.152 d^−1^ represents the global carrying capacity and growth deceleration over the entire 24-day cultivation phase.

### 3.4 Nutrient removal efficiency

#### 3.4.1 Nitrate removal

**FIGURE 6:**
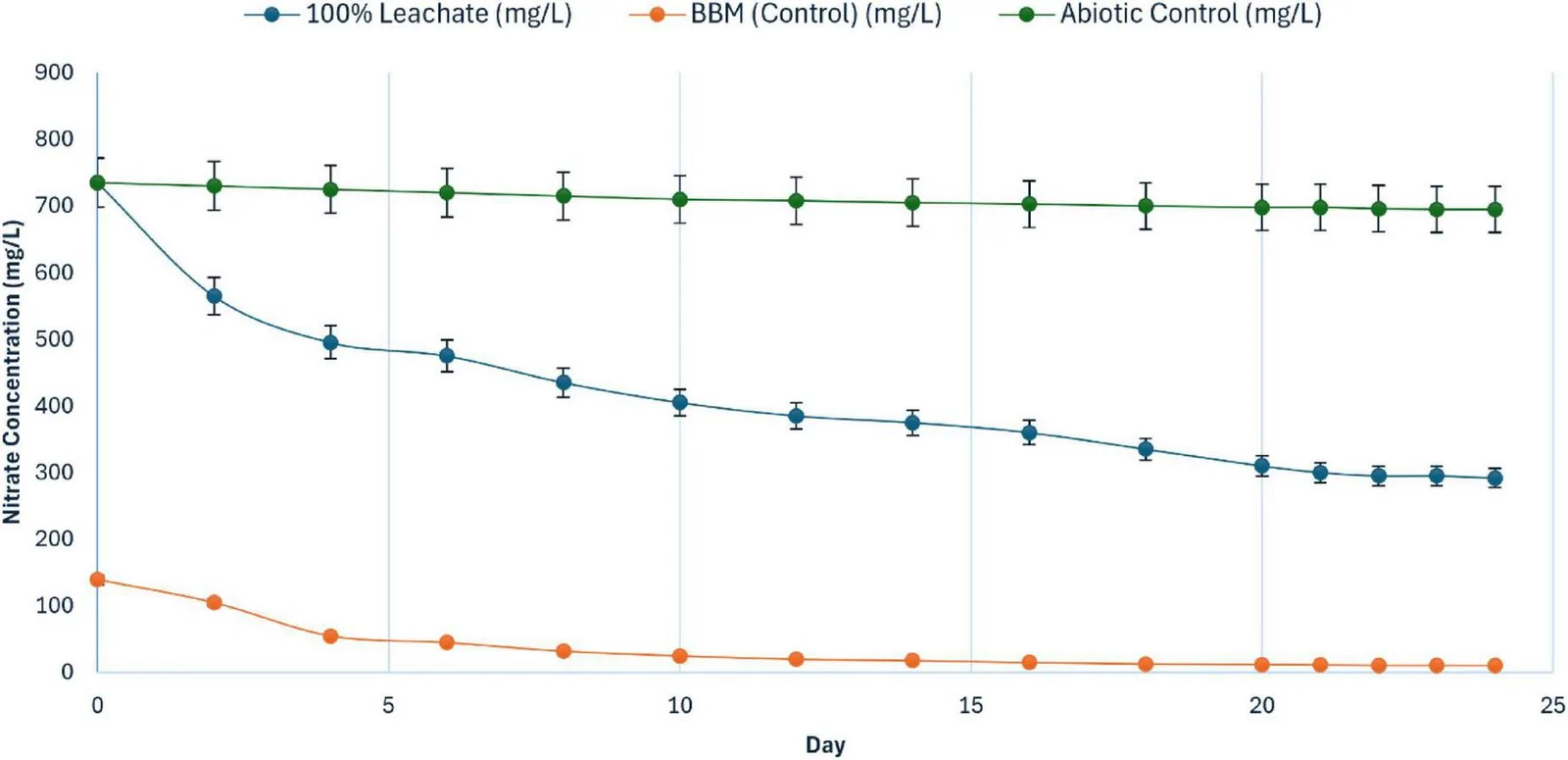
Time-course nitrate removal by *Desmodesmus* sp. from matured compost leachate and the BBM control over the 24-day cultivation period. Error bars represent standard deviation (*n* = 3).

Nitrogen is a critical macronutrient for microalgal protein synthesis and growth. The temporal variation of Nitrate (NO_3_^–^–N) concentration in the matured compost leachate and the BBM control is presented in Figure 6.

The initial nitrate concentration in the matured compost leachate was recorded at 735.29 ± 17.87 mg L^−1^, significantly higher than the synthetic control (139.37 ± 4.42 mg L^−1^), confirming the nitrogen-rich nature of the compost effluent. Despite this high initial load, *Desmodesmus* sp. demonstrated efficient nitrogen uptake capability. A rapid depletion of nitrate was observed during the first 4 days of cultivation, where the nitrate concentration in the leachate dropped sharply to 495.20 ± 16.43 mg L^−1^. Statistical analysis confirms this initial reduction was highly significant (*p* < 0.01), corresponding to a removal of approximately 32.6% of the initial load.

This rapid initial uptake is characteristic of microalgal acclimatization, during which cells rapidly assimilate bioavailable nitrogen to synthesize essential enzymes, chlorophyll, and structural proteins required for the transition into the exponential growth phase. This correlates with the rapid exponential growth phase observed in the biomass data (section 3.2). Following this initial surge, the removal rate stabilized but continued steadily throughout the cultivation period.

By the end of the experiment (day 24), the nitrate concentration in the leachate had decreased to 291.75 ± 12.12 mg L^−1^. This represents a statistically significant total reduction (*p* < 0.001) relative to the initial concentration, achieving a removal efficiency of 60.32%. In comparison, the control medium showed a depletion from 139.37 mg L^−1^ to 10.82 mg L^−1^, achieving a removal efficiency of 92.2%. Crucially, the uninoculated abiotic control exhibited negligible nitrate reduction over the 24-day period ( < 5%), definitively confirming that the observed nitrogen depletion in the experimental cultures was driven by active microalgal assimilation rather than physical precipitation or ammonia volatilization. Although the percentage removal was higher in the control due to the lower initial starting concentration, the total mass of nitrate removed in the leachate system (443.5 mg L^−1^ removed) was substantially higher than in the control (128.5 mg L^−1^ removed). This high nutrient removal capacity highlights the potential of *Desmodesmus* sp. to bioremediate high-strength nitrogenous wastewaters while converting the excess nutrients into valuable biomass.

#### 3.4.2 Phosphate removal

**FIGURE 7:**
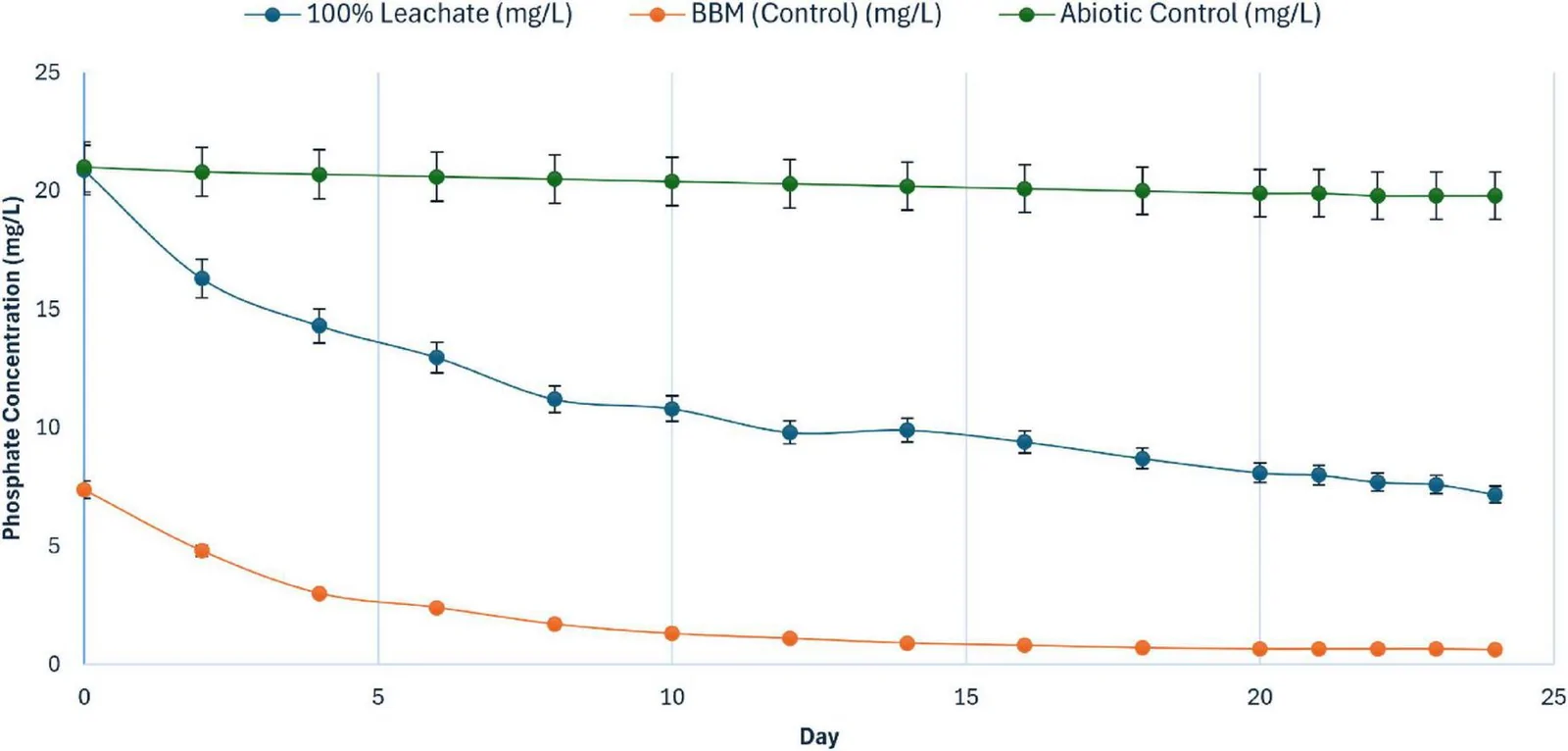
Time-course phosphate removal by *Desmodesmus* sp. from matured compost leachate and the BBM control during the 24-day cultivation. Error bars represent standard deviation (*n* = 3).

Phosphorus is a vital nutrient for microalgal metabolism, primarily involved in energy transfer (ATP synthesis) and nucleic acid formation. The removal of Phosphate (PO_4_^−3^–P) from the matured compost leachate and BBM control was monitored throughout the cultivation period, as shown in Figure 7.

The matured compost leachate initially contained a phosphate concentration of 20.87 ± 1.20 mg L^−1^, which is typical for organic-rich effluents and higher than the control medium (7.37 ± 0.14 mg L^−1^). The removal trend followed a pattern similar to nitrogen uptake, characterized by a rapid reduction during the initial growth phases (days 0–6). During this first week, the phosphate concentration in the leachate decreased to 12.96 ± 0.69 mg L^−1^, indicating active assimilation by the algae during cell division.

By the end of the 24-day cultivation period, the phosphate concentration in the leachate had significantly decreased to 7.18 ± 0.09 mg L^−1^ (*p* < 0.001), corresponding to a removal efficiency of 65.57%. In contrast, the BBM control achieved a higher percentage removal efficiency of 91.54%, with the final concentration dropping to 0.62 ± 0.02 mg L^−1^. Because the experimental culture’s pH exceeded 9.0 (section 3.4.4), the rapid phosphorus removal is a synergistic combination of direct intracellular luxury uptake by the *Desmodesmus* sp. and biologically induced physicochemical precipitation (e.g., calcium phosphate formation). This alkaline chemical precipitation was directly induced by microalgal photosynthetic CO_2_ consumption. The abiotic control remained at pH 6.8 and exhibited negligible phosphorus loss (<5%), verifying that the physicochemical precipitation was strictly biologically mediated.

While the percentage removal was higher in the synthetic control, the absolute mass of phosphorus removed was higher in the wastewater system. *Desmodesmus* sp. removed approximately 13.69 mg L^−1^ of phosphate from the leachate, compared to 6.74 mg L^−1^ in the control. This phosphorus uptake confirms that matured compost leachate provides bioavailable phosphate that can be recovered by microalgal biomass, thereby reducing the eutrophication potential of the effluent before discharge.

#### 3.4.3 Chemical oxygen demand (COD) removal

**FIGURE 8:**
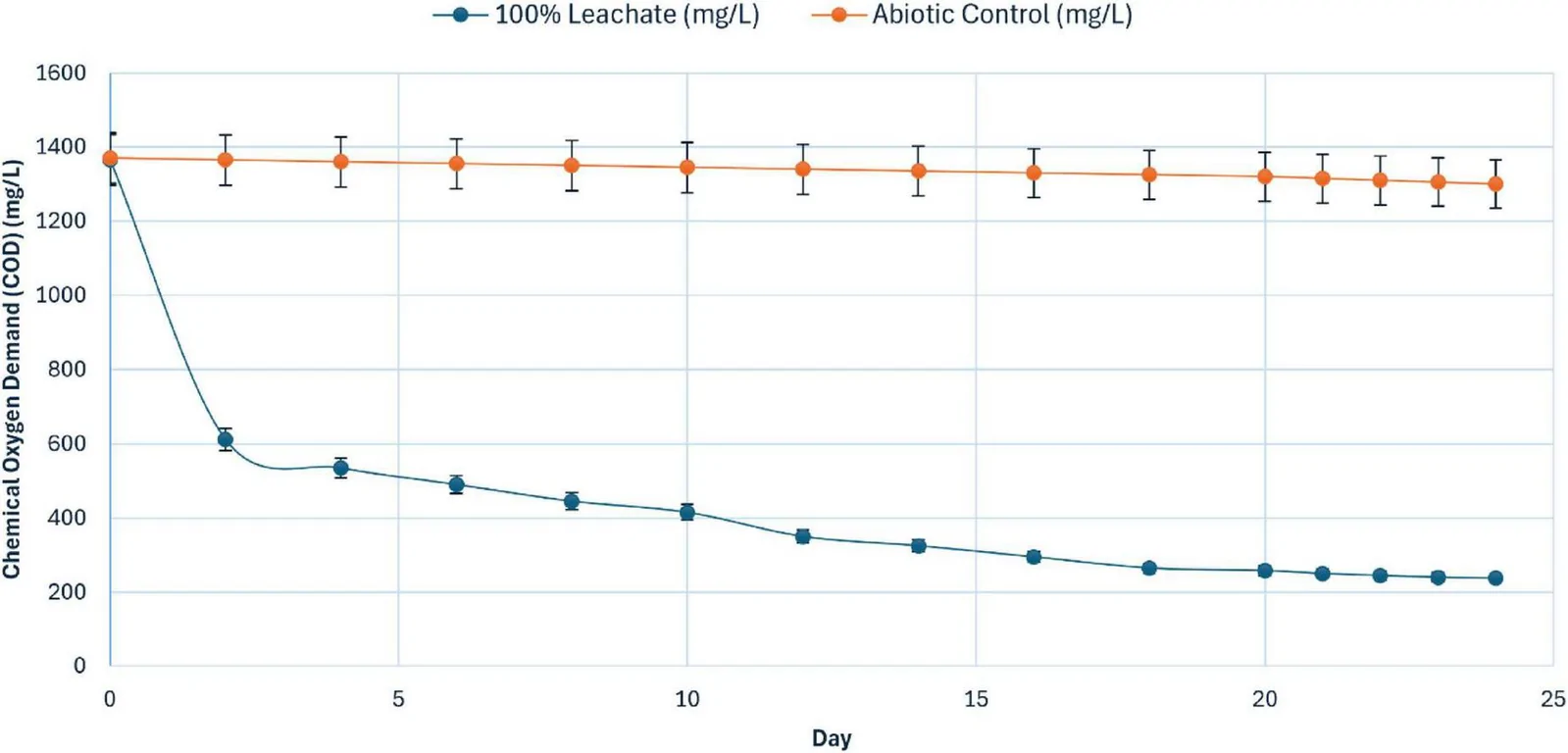
Time-course Chemical Oxygen Demand (COD) reduction in matured compost leachate during cultivation of *Desmodesmus* sp. Error bars represent standard deviation (*n* = 3).

Chemical Oxygen Demand (COD) serves as a primary indicator of the organic matter content in the wastewater. Unlike the inorganic BBM control, which contains negligible organic carbon, the matured compost leachate presented a high initial organic load, necessitating the evaluation of carbon removal efficiency. The temporal reduction of COD in the leachate culture is illustrated in Figure 8.

The initial COD of the matured compost leachate was recorded at 1365.89 ± 48.19 mg L^−1^. A sharp decrease in COD was observed within the first 48 h of cultivation, where the concentration decreased to 611.23 ± 30.48 mg L^−1^. This corresponds to a removal of 55% of the total organic load in just 2 days. This consumption of organic carbon confirms a shift toward mixotrophic metabolism, demonstrating the capacity of *Desmodesmus* sp. to directly assimilate complex organic substrates to fuel the rapid biomass production observed in the early growth phase (section 3.2).

Following this initial assimilation phase, the COD removal rate stabilized, decreasing gradually as the readily biodegradable organic fraction was exhausted. Notably, between days 20 and 24, the COD concentration remained relatively constant. During this late stationary phase, the microalgae transitioned away from mixotrophic organic carbon consumption, relying instead on autotrophic carbon fixation and the intracellular reallocation of carbon fluxes toward lipid biosynthesis, driven by the exhaustion of available phosphorus.

By day 24, the final COD concentration reached 237.43 ± 10.95 mg L^−1^, representing a statistically significant reduction (*p* < 0.001) from the initial value. The overall COD removal efficiency achieved was 82.62%. Furthermore, the abiotic control exhibited minimal COD reduction ( < 5%) over the 24-day period, confirming that the organic carbon depletion was driven by active microalgal assimilation rather than physical settling or volatilization. This removal efficiency indicates that the pure mixotrophic metabolism of *Desmodesmus* sp. is effective at mineralizing the organic pollutants present in matured compost leachate, demonstrating the dual capability of this system for both biomass generation and organic wastewater treatment. This depletion of external organic carbon may have further contributed to the metabolic shift toward intracellular carbon reallocation and lipid accumulation observed during the stationary phase.

#### 3.4.4 pH evolution during cultivation

**FIGURE 9:**
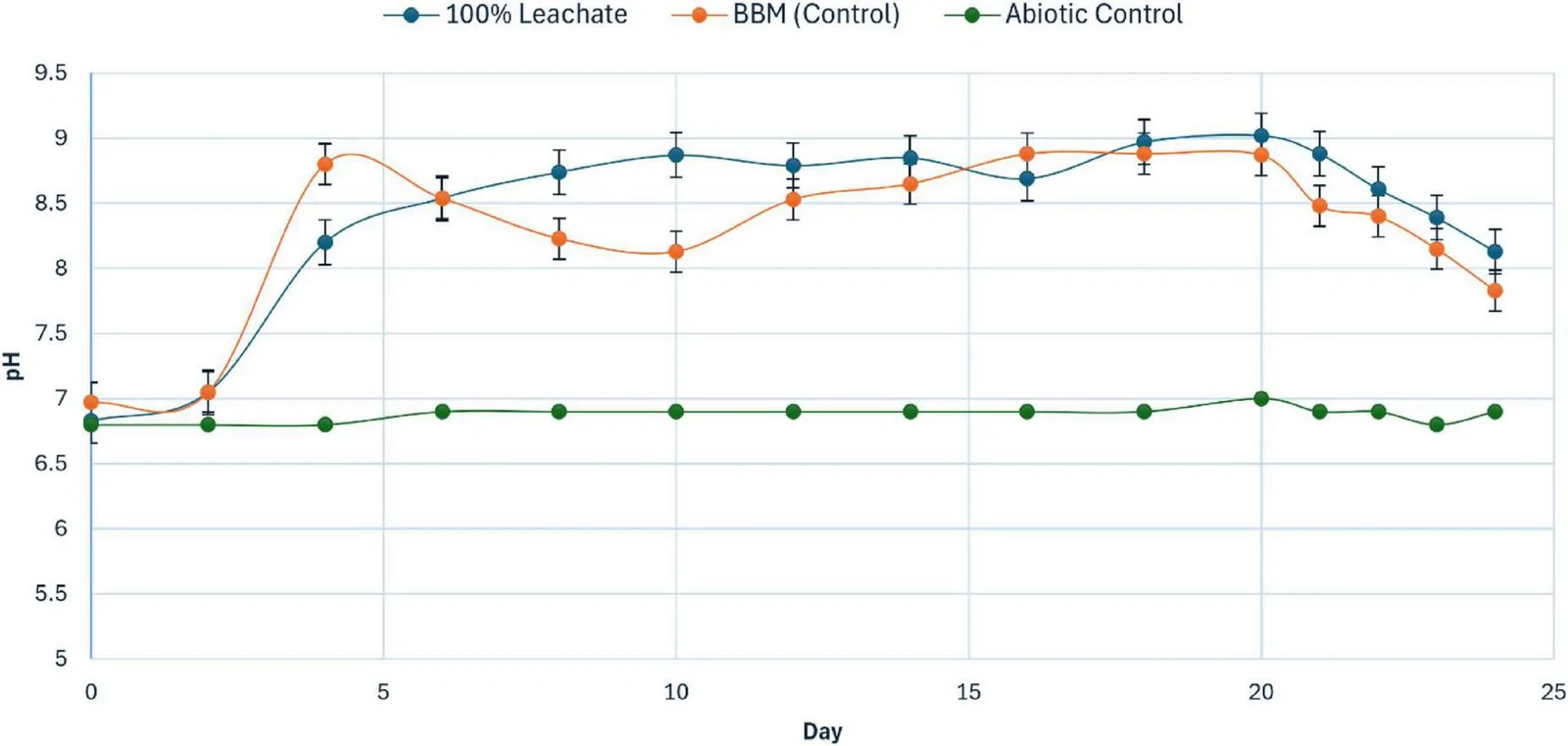
pH evolution of matured compost leachate and the BBM control during the cultivation of *Desmodesmus* sp. Error bars represent standard deviation (*n* = 3).

The variation of pH in the culture medium serves as an indirect proxy for microalgal metabolic activity, particularly photosynthetic carbon fixation. The pH profiles of the matured compost leachate and control cultures are presented in Figure 9.

Both media started with a near-neutral pH (6.83 ± 0.15 for leachate and 6.97 ± 0.18 for control). As cultivation progressed, an alkalinization trend was observed in both conditions. In the matured compost leachate, the pH rose, reaching a maximum value of 9.02 ± 0.18 by day 20. A similar trend was observed in the control, which peaked at 8.88 ± 0.18. In contrast, the uninoculated abiotic control maintained a relatively stable pH (fluctuating marginally around 6.8–7.0) throughout the 24-day period. This supports the conclusion that the alkalinization observed in the experimental cultures was biologically mediated.

This increase in pH is attributed to the photosynthetic consumption of dissolved inorganic carbon. As *Desmodesmus* sp. actively assimilates dissolved CO_2_ and bicarbonates (HCO_3_^–^) during the light period, the equilibrium of the carbonate system shifts, leading to the consumption of hydrogen ions (H^+^) and a subsequent rise in hydroxyl ions (OH^–^). The fact that the pH remained consistently high ( > 8.5) from days 6 to 20 indicates that the culture was maintaining active photosynthesis throughout the exponential growth phase.

In highly turbid, mixed bioreactors containing undiluted leachate, light is rapidly attenuated, raising the paradox of how active photosynthesis is maintained. In these fluid dynamics, a narrow “photic zone” exists at the reactor surface and near the illuminated walls. As cells circulate into this photic zone, they perform rapid photosynthesis (driving up the pH through dissolved inorganic carbon consumption), while cells in the darker bulk volume rely on mixotrophic and heterotrophic assimilation.

Towards the end of the cultivation (days 22–24), a decline in pH was observed (dropping to 8.13 in leachate), signaling the onset of the stationary phase where cellular respiration and organic acid release began to counterbalance photosynthetic carbon uptake.

### 3.5 Lipid production and accumulation

**FIGURE 10:**
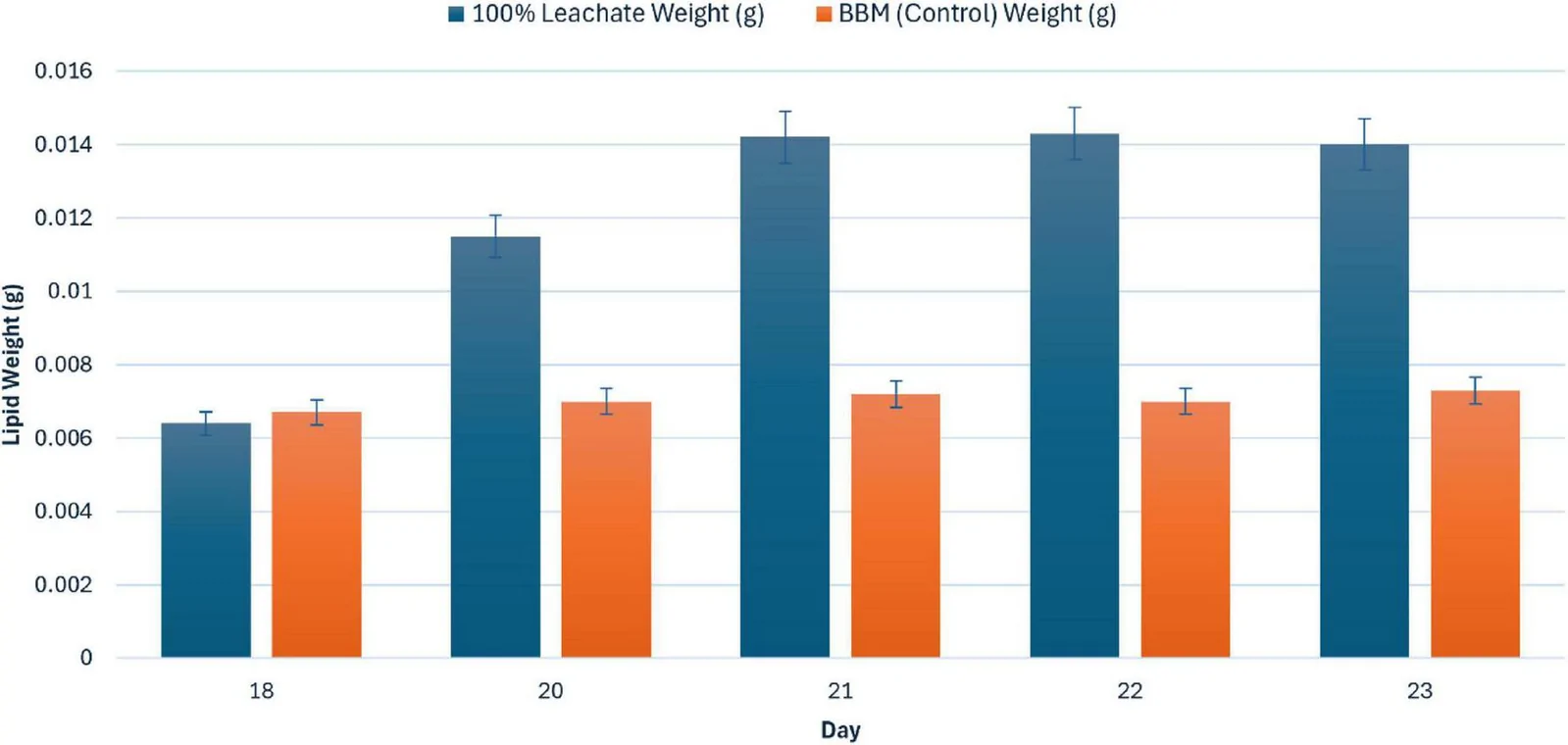
Lipid accumulation (g) of *Desmodesmus* sp. in matured compost leachate and the BBM control from days 18 to 23. Error bars represent standard deviation (*n* = 3).

**FIGURE 11:**
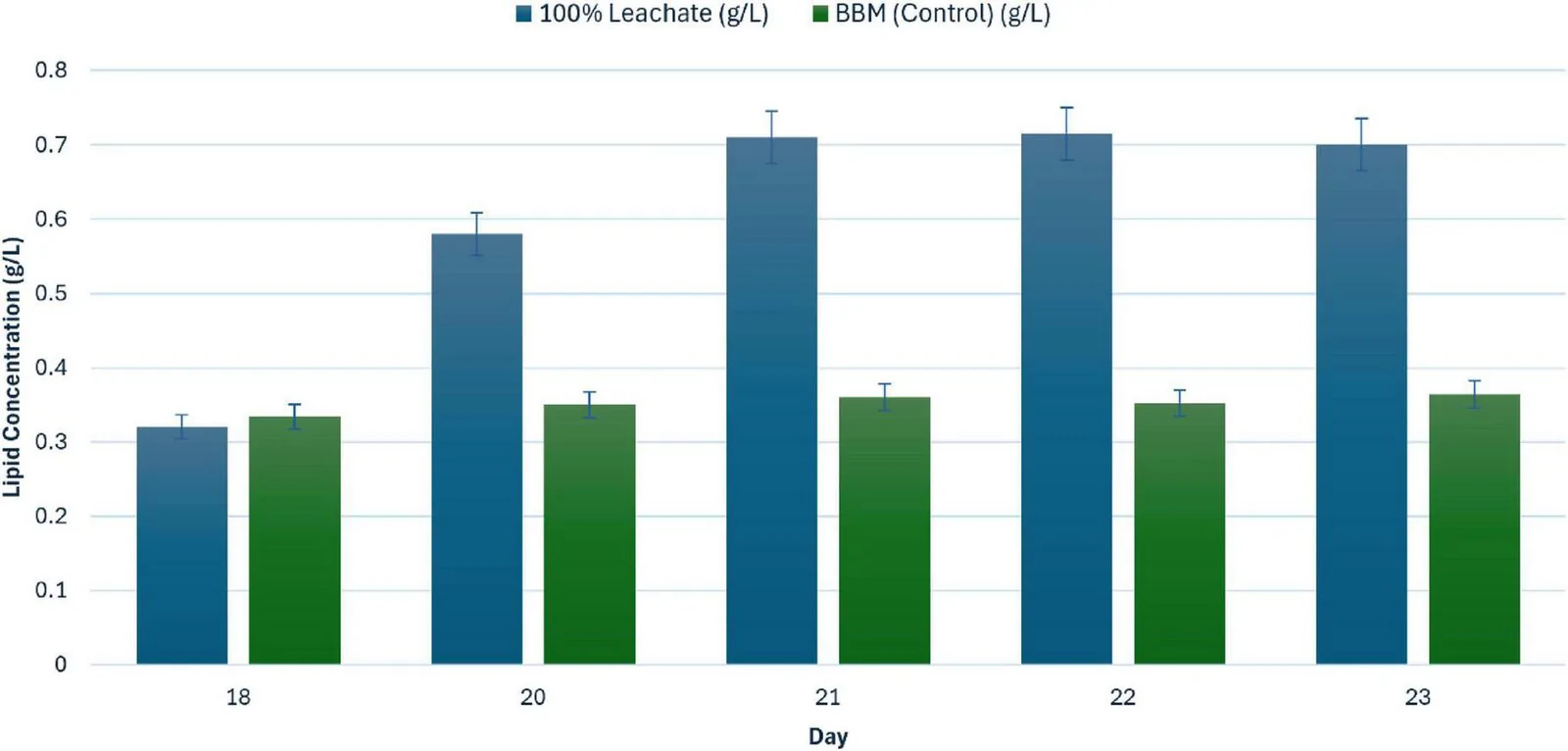
Lipid concentration (g L^−1^) of *Desmodesmus* sp. in matured compost leachate versus the BBM control during the stationary phase (days 18–23). Error bars represent standard deviation (*n* = 3).

The viability of microalgal biodiesel depends not only on biomass growth but critically on the lipid yield per unit volume of culture. To evaluate this, lipid weight (g) and lipid concentration (g L^−1^) were quantified during the transition from the exponential to the stationary phase (days 18–23), as presented in Figures 10, 11.

At day 18, the lipid concentration in the matured compost leachate (0.320 ± 0.002 g L^−1^) remained comparable to the BBM control (0.334 ± 0.009 g L^−1^). This indicates that during the active growth phase, metabolic energy was primarily directed toward cellular division and biomass synthesis rather than lipid storage.

However, a metabolic shift occurred as the culture transitioned into the stationary phase. In the matured compost leachate cultures, the lipid concentration increased between days 18 and 22. The lipid yield more than doubled during this 4-day window, peaking at a maximum concentration of 0.715 ± 0.027 g L^−1^ on day 22. Expressing this yield as a percentage of dry cell weight (% DCW) reveals an intracellular lipid accumulation reaching 26.56% DCW in the leachate system.

While the leachate retained 291.75 mg L^−1^ of nitrate at the end of the cultivation (ruling out nitrogen starvation), the initial highly skewed N:P ratio (∼35:1) led to a rapid and near-complete exhaustion of available phosphorus (section 3.4.2). This rapid lipid accumulation, coinciding directly with phosphorus depletion and potential light limitation within the turbid matrix, aligns with established microalgal stress responses. Under severe phosphorus exhaustion, *Desmodesmus* sp. redirects carbon flux away from cell division and toward triglyceride (TAG) storage. Furthermore, during this stationary phase, as the assimilation of external organic carbon (COD) leveled off, the cells relied on the intracellular reallocation of existing carbon reserves to drive lipid biosynthesis (Abrha et al., 2025; Bonnefond et al., 2017; Liufu et al., 2025; Rios et al., 2015; Zhu et al., 2022).

In contrast, the control cultures displayed a stagnant lipid profile, with concentrations fluctuating marginally around 0.350–0.360 g L^−1^ throughout the stationary phase. Consequently, by day 22, the matured compost leachate yielded approximately 2.03 times more lipid per liter compared to the synthetic control. This behavior differs from conventional synthetic media-based cultivation, highlighting the unique metabolic response induced by compost leachate. This superior volumetric lipid productivity confirms that matured compost leachate not only supports higher biomass densities but effectively triggers lipid accumulation, making it an excellent feedstock for scalable biodiesel production.

### 3.6 Fatty acid methyl ester (FAME) profiling

The quality and performance of microalgal biodiesel are governed by the fatty acid profile of the parent oil. The FAME composition of *Desmodesmus* sp. cultivated in matured compost leachate was determined via GC-FID analysis (Table 3).

**TABLE 3:** Fatty acid profile of *Desmodesmus* sp. biomass cultivated in matured compost leachate (normalized % of total FAMEs).

Fatty acid	Carbon structure	Relative abundance (%)
Caprylic acid	C8:0	4.55
Capric acid	C10:0	3.86
Lauric acid	C12:0	25.78
Myristic acid	C14:0	9.39
Palmitic acid	C16:0	26.95
Stearic acid	C18:0	5.11
Oleic acid	C18:1	20.46
Linoleic acid	C18:2	3.90
Total saturated	–	75.64
Total unsaturated	–	24.36

The raw chromatograms indicated that the crude lipid extract contained non-saponifiable lipids, primarily stigmasterol (RT 29.24 min), which accounted for 38% of the raw peak area. While the normalized FAME profile determines the theoretical fuel properties, the presence of this non-saponifiable fraction reduces the overall conversion efficiency of the crude extract into usable biodiesel and requires accounting in practical assessments. For biodiesel characterization, the profile was normalized to include only fatty acid methyl esters. The analysis demonstrated a profile comprising primarily saturated fatty acids (SFAs), specifically medium-chain fatty acids. The most abundant components were palmitic acid (C16:0) at 26.95% and lauric acid (C12:0) at 25.78%. The presence of short-chain fatty acids such as caprylic (C8:0) and capric (C10:0) acids was detected, which influences the fuel’s flow properties.

This profile, characterized by shorter, saturated chains (C12–C16), contrasts with baseline synthetic media cultivation. Based on the standard BBM control data (150 μmol photons m^–1^ s^−1^, N-sufficient), the baseline FAME profile is highly unsaturated, dominated by γ-linolenic acid (C18:3, ∼63.2%), with palmitic acid (C16:0) comprising only ∼5.1%. The observed enrichment of medium-chain saturated fatty acids (75.64% total SFAs) in the leachate-grown biomass confirms a definitive metabolic shift. This indicates a stress response to the complex organic matrix and phosphorus exhaustion of the compost leachate, favoring reduced carbon chain elongation and desaturation under these specific cultivation conditions.

## 4 Discussion

The ability of *Desmodesmus* sp. to sustain growth in 100% matured compost leachate without dilution represents an advance in wastewater-integrated microalgal cultivation. High-strength organic effluents are characterized by elevated nitrogen and organic loads that often necessitate dilution or pre-treatment. Unlike fresh leachates, the matured compost leachate used in this work exhibited greater chemical stability and a balanced organic-nutrient profile. These characteristics mitigated the inhibitory effects typically observed at high organic loads, enabling sustained growth under undiluted conditions (Ji et al., 2014; Abdelfattah et al., 2023; Liew et al., 2025).

Sustained pure-culture cultivation of *Desmodesmus* sp. in 100% sterilized matured compost leachate without dilution has not been widely reported. While the 75% dilution yielded the highest absolute dry biomass during the screening phase (section 3.2), the 100% undiluted leachate was selected for the main cultivation phase to evaluate the feasibility of a zero-dilution process, which offers operational and water conservation advantages for large-scale wastewater integration.

Within this context, the 4-day lag phase observed under undiluted conditions functions as a physiological acclimatization period. Cells pre-cultured in photoautotrophic synthetic media require time to activate the enzymatic pathways necessary for mixotrophic assimilation of complex organics in a dark, turbid matrix. During this period, background toxicity from complex organics and free ammonia (NH3) can disrupt photosystem II and uncouple electron transport, causing transient photosynthetic suppression (Markou et al., 2016; Drath et al., 2008). In response, cells activate detoxification and nitrogen assimilation pathways, such as the ATP-dependent GS/GOGAT cycle, to convert toxic nitrogen species into structural biomass. The subsequent recovery confirms metabolic restructuring, supporting the feasibility of gradual acclimatization strategies for high-strength effluents (Ji et al., 2014; Abdelfattah et al., 2023; Guo et al., 2025; Wang et al., 2019).

Following acclimatization, rapid biomass accumulation demonstrates the energetic advantage of mixotrophic metabolism. Mixotrophy enables the simultaneous utilization of light energy and organic carbon (COD), promoting energetic coupling between chloroplasts and mitochondria (Abdelfattah et al., 2023; Yan et al., 2024; Zhang et al., 2021; Bo et al., 2021; Tossavainen et al., 2017). Consequently, *Desmodesmus* sp. exploits the biodegradable organic matter present in the leachate, overcoming photosynthetic limitations and explaining the 2.69 g L^−1^ biomass yield (Abdelfattah et al., 2023; Eze et al., 2018; Cheng et al., 2013; de Souza et al., 2021; Yun et al., 2021). Furthermore, the transient declines observed in the specific growth rate profile (Figure 5) reflect diauxic shifts as the microalgae sequentially transition between the assimilation of bioavailable low-molecular-weight organics and more recalcitrant carbon sources (Abdelfattah et al., 2023; Yun et al., 2021).

Sustained growth in optically dense leachate was achieved despite the background turbidity. In conventional photobioreactors, turbidity restricts the photic zone and induces photolimitation (Abdelfattah et al., 2023; Segredo-Morales et al., 2023; Otim et al., 2021; Benner et al., 2022). Under mixotrophic regimes, organic turbidity provides a functional advantage, as particulate and dissolved organic compounds serve as metabolizable carbon sources. By reducing reliance on photon penetration within the dark bulk volume of the reactor, *Desmodesmus* sp. sustained growth, effectively bypassing classical shadowing effects (Abdelfattah et al., 2023; Wu et al., 2025; Licata et al., 2025).

The 82.6% COD removal efficiency demonstrates a capacity to remediate matured compost leachate, exceeding the 36-70% reductions reported for high-strength effluents (Abdelfattah et al., 2023; Ahmed et al., 2017). This attenuation is facilitated by the leachate’s composition, which is dominated by readily bioavailable, low-molecular-weight compounds (Abdelfattah et al., 2023; Debeni Devi et al., 2022; Doyle et al., 2022). The utilization of a strict abiotic control confirms that COD removal in this system was driven directly by the mixotrophic assimilation pathways of *Desmodesmus* sp., rather than bacterial oxidation.

Nitrate removal reached 60.3%, indicating nitrogen attenuation despite the initial concentration (∼735 mg L^−1^). Nitrogen removal is driven by biological assimilation into cellular components (Song et al., 2024; Salbitani and Carfagna, 2021). However, the initial N:P ratio (∼35:1) established an extreme stoichiometric imbalance within the leachate matrix. Recent systematic evaluations in *Spirulina platensis* demonstrate that specific N/P stoichiometry acts as a primary metabolic switch; when microalgae are exposed to high absolute nitrogen concentrations but severe relative phosphorus limitation, the culture experiences metabolic stress that aggressively suppresses biomass growth and redirects carbon flux toward lipid biosynthesis (Barahoei et al., 2026; Bhattacharjee and Baruah, 2025; Bhattacharjee and Baruah, 2026).

This mechanistic principle directly explains the lipid accumulation observed in this study. Because the leachate retained 291.75 mg L^−1^ of nitrate at the end of the cultivation phase, classical nitrogen starvation was not the inductive trigger. Instead, the rapid exhaustion of available phosphorus (dropping to 7.18 mg L^−1^) induced a metabolic shift. This rapid phosphorus depletion was achieved via a synergistic combination of direct intracellular luxury uptake and biologically induced physicochemical precipitation, driven by microalgal photosynthetic CO_2_ consumption and the resulting alkaline shift (pH > 9.0). The onset of this phosphorus exhaustion coincided directly with the lipid accumulation phase (yielding 0.715 g L^−1^). The high COD environment sustained carbon availability, enabling high triglyceride (TAG) productivity without the need to artificially induce nitrogen starvation (Abdelfattah et al., 2023; Yun et al., 2021; Apandi et al., 2020; Bomer and Leverett, 2024; Sriram and Seenivasan, 2015).

This phosphorus exhaustion and complex organic matrix also induced a definitive shift in the fatty acid profile. While baseline cultivation in synthetic BBM yields a highly unsaturated profile dominated by C18:3 (∼63.2%), the leachate-grown cells yielded a lipid profile comprising 75.6% saturated fatty acids (SFAs), dominated by palmitic acid (C16:0) and lauric acid (C12:0) (Vimali et al., 2022). Such profiles support the “membrane hardening” hypothesis, whereby reduced carbon chain elongation and desaturation enhance cellular stability under adverse matrix conditions and severe phosphorus limitation (Abdelfattah et al., 2023; De Bhowmick et al., 2025; Chen et al., 2022; Arguelles et al., 2019).

It is necessary to acknowledge that the FAME analysis identified quantities of non-saponifiable lipids, primarily stigmasterol, in the raw extract. In future practical applications, the presence of this fraction must be accounted for, as it influences the overall conversion efficiency of the crude extract during transesterification.

From a sustainability perspective, the direct cultivation of *Desmodesmus* sp. in matured compost leachate eliminates the need for synthetic nutrients, reducing operational costs. Simultaneous nutrient recovery, carbon utilization, and the avoidance of freshwater dilution position this approach within a circular bioeconomy framework. Future research should focus on pilot-scale validation, reactor optimization, and integrated techno-economic assessment to facilitate practical implementation.

## 5 Conclusion

This study confirms the feasibility of utilizing undiluted matured compost leachate as a sole, low-cost substrate for the mixotrophic cultivation of *Desmodesmus* sp., effectively eliminating reliance on synthetic nutrient inputs. The strain exhibited metabolic adaptability, achieving a maximum biomass concentration of 2.69 g L^−1^—approximately fourfold higher than the synthetic control, while simultaneously enabling efficient wastewater remediation, with 82.6% COD removal, 60.3% nitrate reduction, and 65.5% phosphate removal. The inclusion of a sterile abiotic control verified that COD and nitrate removal were driven by direct microalgal assimilation, while phosphate depletion resulted from a synergistic combination of biological uptake and photosynthesis-induced physicochemical precipitation.

The intrinsic nutrient profile of the leachate enabled a self-regulating two-stage metabolic response, where progressive phosphorus exhaustion (driven by the extreme ∼35:1 initial N:P ratio) during the stationary phase was associated with a shift in carbon flux toward lipid biosynthesis. This resulted in a lipid yield of 0.715 g L^−1^ (26.56% DCW). The corresponding FAME profile was dominated by saturated medium-chain fatty acids (75.6%), indicating oxidative stability and cetane characteristics suitable for biodiesel applications.

However, key limitations must be addressed for industrial translation. The high saturation level may constrain cold-flow performance, necessitating blending or additive strategies. In addition, the presence of a non-saponifiable lipid fraction (predominantly stigmasterol) represents a bottleneck that could reduce transesterification efficiency if not properly managed.

Finally, this work demonstrates a zero-dilution, waste-to-resource platform that integrates high-density biomass production with effective nutrient recovery. Future research should focus on pilot-scale continuous cultivation and engine-level fuel validation to bridge the gap between laboratory performance and real-world deployment.

## Data Availability

The original contributions presented in this study are included in the article/supplementary material, further inquiries can be directed to the corresponding authors.
